# Study Hotspot and Trend in the Field of Shear Wave Elastography: A Bibliometric Analysis from 2004 to 2024

**DOI:** 10.2174/0115734056353590250109081225

**Published:** 2025-01-15

**Authors:** Jingjing Zhao, Linping Pian, Jie Chen, Quanjiang Wang, Feiyan Han, Yameng Liu

**Affiliations:** 1 Department of Ultrasound Medical, Henan University of Traditional Chinese Medicine, Zhengzhou, China; 2 Department of Ultrasound Medical, The First Affiliated Hospital of Henan University of Traditional Chinese Medicine, Zhengzhou, China

**Keywords:** Shear wave elastography, Bibliometrics, Visualized analysis, Contrast-enhanced ultrasound, Multimodal ultrasound, Transient elastography

## Abstract

**Background:**

The objective of this study was to comprehensively review the literature on Shear Wave Elastography (SWE), a non-invasive imaging technique prevalent in medical ultrasound. SWE is instrumental in assessing superficial glandular tissues, abdominal organs, tendons, joints, carotid vessels, and peripheral nerve tissues, among others. By employing bibliometric analysis, we aimed to encapsulate the scholarly contributions over the past two decades, identifying key research areas and tracing the evolutionary trajectory of SWE.

**Methods:**

For this study, we selected research articles related to SWE published between 2004 and March 2024 from the Web of Science Core Collection (WOSCC). We utilized sophisticated bibliometric tools, such as CiteSpace, VOSviewer, and SCImago Graphica, to analyze the trends in annual publications, contributing countries and institutions, journals, authors, co-cited authors, co-cited references, and keywords.

**Results:**

Our analysis yielded a total of 3606 papers. China emerged as the leading country in terms of publication output, with a strong collaborative relationship with the United States. Sun Yat-Sen University was identified as the institution with the highest number of publications. The keyword “transient elastography” was the most prevalent, with “acoustic radiation force” being a focal point in the initial stages of SWE research. Recently, Contrast-enhanced Ultrasound (CEUS) has emerged as a new research focus, signaling a potential direction for future research and development.

**Conclusion:**

The global research landscape for SWE is projected to expand continuously. Future research is likely to concentrate on the integrated application of SWE and CEUS for diagnostic purposes, along with exploring the clinical utility of multimodal ultrasound that synergistically combines SWE with other ultrasound technologies. This bibliometric research offers a comprehensive overview of the SWE literature, guiding researchers in their pursuit of further exploration and discovery.

## 1 INTRODUCTION

SWE represents a significant advancement in non-invasive imaging technology, enabling the quantification of tissue stiffness. This technique employs an ultrasonic probe to generate acoustic radiation force, which induces shear waves within the target tissue. The resulting vibration is captured using ultrafast ultrasonic imaging, allowing for the real-time visualization of tissue hardness through a color-coded representation [1]. SWE stands out from strain elastic imaging due to its superior objectivity, accuracy, and repeatability, independent of external pressure [2].

In 2011, the European Federation of Ultrasound in Medicine and Biology (EFSUMB) established guidelines for the clinical use of elastography, marking a pivotal moment in the development of SWE [1]. Since then, this technique has experienced rapid growth, with its clinical applications expanding exponentially, capturing the interest of numerous researchers. SWE has been increasingly utilized in the assessment of superficial glandular tissues, abdominal organs, tendons, joints, carotid vessels, and peripheral nerve tissues, offering significant contributions to the classification of diffuse lesions, the differential diagnosis of focal lesions, disease monitoring, and follow-up [3-5].

Bibliometrics, the quantitative analysis of literature through mathematical and statistical methods, extends beyond simple publication counts. It encompasses the examination of collaborative efforts across nations, authors, and institutions, as well as the identification of frequently occurring keywords [6, 7]. By leveraging tools, like CiteSpace, VOSviewer, and SCImago Graphica, this study aimed to conduct a statistical and visual analysis of the relevant SWE literature. This approach can facilitate the identification of current research hotspots, trends, and potential future directions [8-10]. In doing so, we seek to contribute to the generation of new ideas and insights within the field of SWE.

## 2 MATERIALS AND METHODS

### 2.1 Research Strategy and Data Acquisition

The research strategy for this study was meticulously designed to ensure comprehensive coverage of the literature on SWE. The SCI-E of the WOSCC was chosen as the primary database due to its extensive coverage of over 20,000 peer-reviewed journals across various disciplines. This platform’s unique capability to curate citing references for each indexed publication made it ideal for conducting co-citation analysis, a critical component of bibliometric research [11].

Utilizing the SCI-E database within the WOSCC, a thorough search was conducted, spanning from January 1, 2004, to March 31, 2024. To mitigate any potential bias introduced by the database’s update schedule, the search was finalized on March 31, 2024, to capture the most recent publications. The search strategy was constructed to target articles and reviews related to SWE, using the following search terms: (TS= (“ultrasonography shear wave” OR “sonography shear wave” OR “ultrasonic shear wave” OR “ultrasound shear wave” OR “shear wave elastography” OR “shear wave elasticity imaging”)). The search was limited to English-language articles and reviews, excluding other formats, such as meeting abstracts, letters, and unpublished works in languages other than English.

**Fig. (1):**
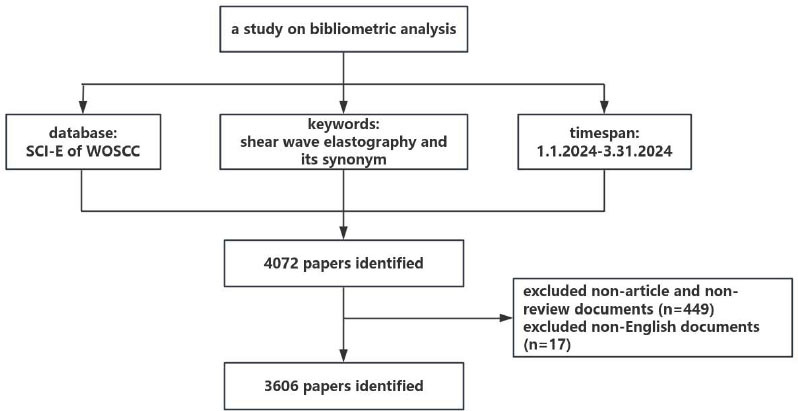
Detailed flowchart of research study.

Following the initial search, three researchers independently reviewed the full text of the retrieved articles to exclude those that were deemed unrelated to the research topic. The researchers excluded the studies primarily focused on non-SWE ultrasound techniques (*e.g*., Doppler or traditional B-mode ultrasound). In cases of disagreement, discussions and consultations among the researchers were used to resolve conflicts. If disputes persisted, it was recommended to involve a third reviewer or re-evaluate the disputed articles to ensure consistency and objectivity in the inclusion process. This rigorous selection process resulted in the identification of a total of 3,606 papers, comprising 3,346 articles and 260 reviews, as the primary dataset for this study (Fig. **1**).

### 2.2 Bibliometric Analysis

The relevant papers retrieved from the SCI-E database in WOSCC were exported as “plain text file”; the export format included “full recorded and cited references”, the files were saved in “download-txt” format, the timespan was set from January 2004 to March 2024, and the time slice was 1 year. Finally, CiteSpace 6.3, VOSviewer 1.6.18, and Bibliometrix 4.2.0 R-package were used to analyze keywords, authors, countries, periodicals, institutions, and co-citations of literature and generate charts.

To analyze the collected data, we employed a suite of advanced bibliometric tools, including CiteSpace 6.3, VOSviewer 1.6.18, and SCImago Graphica 1.0.35. These tools were instrumental in processing the vast array of keywords, identifying the countries and institutions that have contributed significantly to SWE research, analyzing the impact of various journals, profiling the key authors and their co-citations, and examining the co-cited references and keywords. Through the application of these tools, we were able to generate a series of charts and diagrams that visually represented the findings of our analysis, providing a comprehensive overview of the SWE research landscape. This study included a bibliometrics analysis, which analyzed only published literature and did not involve patients. Therefore, ethics approval and consent to participate were not applicable to this study.

## 3 RESULTS

### 3.1 Annual Publications and Trends

**Fig. (2):**
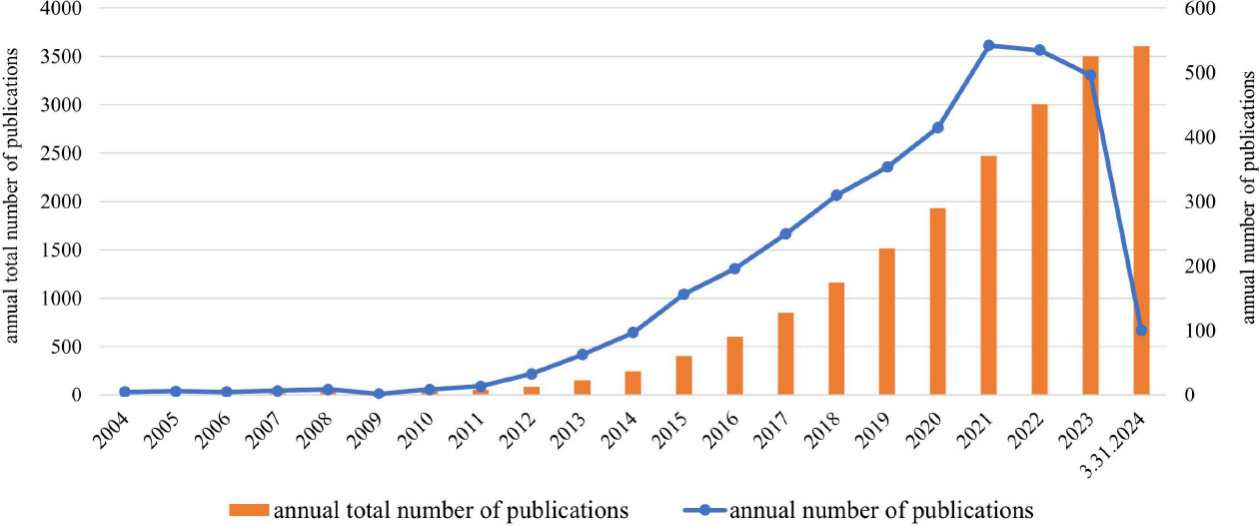
Annual publications and trends.

The study examined the annual publication volume for the 3,606 papers included in the analysis (Fig. **2**). A notable shift occurred in 2011; this surge can be attributed to the introduction of the EFSUMB guidelines for elastography. This pivotal event likely contributed to the subsequent surge in publications, with the number of papers steadily rising year after year. The period between 2020 and 2021 witnessed an exponential increase in published papers, culminating in a peak in 2021. The overall upward trend remained intact, even though there may be temporary fluctuations in the rate of research output. It is important to note that the study’s completion before the end of 2024 may have excluded some recently published articles, which could partially explain the observed decline in 2024.

### 3.2 Distribution of Countries and Institutions

**Fig. (3):**
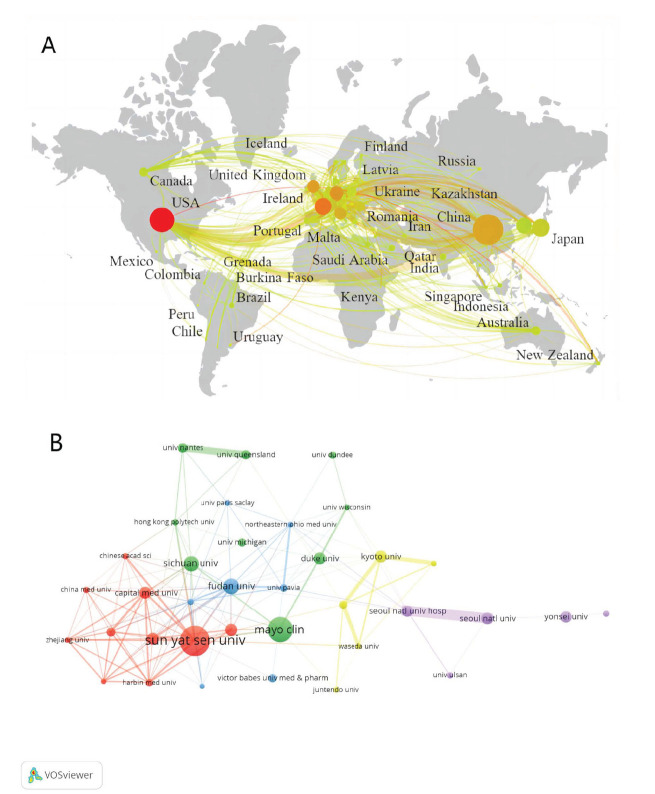
Collaboration networks between countries and institutions. (**A**) Collaboration networks between countries. (**B**) Collaboration networks between institutions. The node size indicates the number of publications; the link size refers to the cooperation intensity. The same color indicates a strong correlation among the literature published by the institutions.

The study encompassed research contributions from a global network of 70 countries. The countries were determined based on the affiliations of all authors listed in the publications, not limited to the first author. Among these, China stood out as the leading country in terms of publications (n=993), followed by the United States (n=645) and Japan (n=340) (Fig. **3A**). Centrality represents the importance and influence of a node in a network. The United States, with a centrality score of 0.49, was not only the highest centrality of publication output, but also held the highest number of citations (16,745) (Table **1**). Total Link Strength (TLS) represents the sum of all connections between a node and other nodes, and it reflects the degree of closeness in the relationships between nodes. The data showed that the United States cooperated most with other countries and had the strongest Total Link Strength (TLS), with China being the closest.

A total of 3053 institutions contributed to the field (Fig. **3B**). The top 10 institutions, as detailed in Table **2**, were predominantly from China and the United States, with Sun Yat-Sen University in China leading the pack with 130 publications, followed by Mayo Clinic (n=108) and Fudan University (n=67). Notably, the top three most-cited institutions were Mayo Clinic (2961), Sun Yat-Sen University (2578), and Duke University (1764), respectively. The visualization of collaboration networks revealed that high-yield institutions engaged in extensive cooperation with other institutions.

### 3.3 Distribution of Journals

**Fig. (4):**
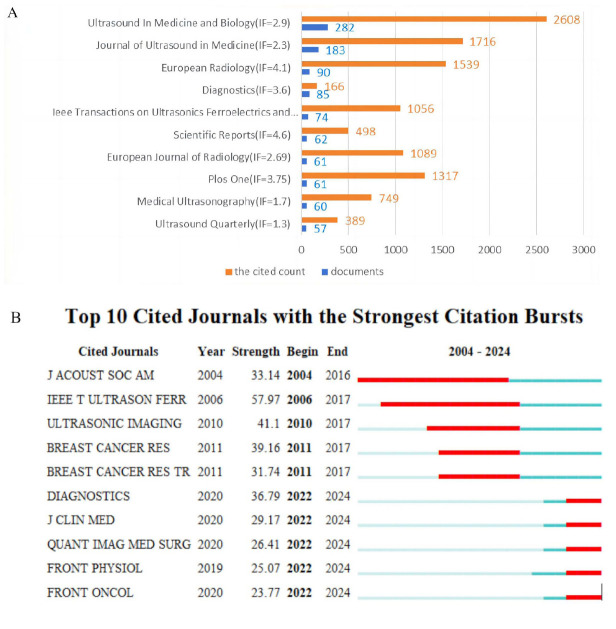
Visualization of journal analysis. (**A**) Top 10 most productive journals. Blue represents the documents of the journal, and yellow represents the cited count. (**B**) Top 10 journals with the strongest citation bursts on SWE research. The red bar represents the most frequently cited journals during this period, while the green bar indicates that the journal was less frequently cited for the rest of the year. “Year” represents the year when the journal began publishing relevant literature. “Start” and “End” indicate the appearance and disappearance of the red bars, representing the period during which the journal was frequently cited in this field. “Strength” quantifies the frequency of citations the journal received during that time period.

Over the course of the study period from 2004 to 2024, SWE research papers were disseminated across a total of 752 journals. Among these, scientific reports from the United Kingdom held the distinction of having the highest Impact Factor (IF) of 4.6. However, in terms of sheer publication volume, Ultrasound in Medicine and Biology, also based in the United Kingdom, led with 282 papers, boasting an IF of 2.9. This journal also held the record for the most citations, with 2608, as depicted in Fig. (**4A**). To identify the journals with the strongest citation bursts, we compiled a list of the top 10 journals in this category (Fig. **4B**). Notably, the Journal of The Acoustical Society of America was frequently cited early on and maintained its presence in the citation landscape for an extended period. More recently, SWE research has been primarily published in five journals: Diagnostics, Journal of Clinical Medicine, Quantitative Imaging in Medicine and Surgery, Frontiers in Physiology, and Frontiers in Oncology. These publications have been found to provide a valuable resource for scholars looking to submit their work in related fields.

### 3.4 Distribution of Authors

The study encompassed the contributions of 14,031 authors. Among these authors, Ioan Sporea from Romania stood out with the highest number of publications (n=51), demonstrating a high level of productivity and collaboration with other authors. Close behind were Matthew W. Urban from the United States (n=50) and Mickaël Tanter from France (n=42) (Table **3**). The visualization of author collaboration in Fig. (**5**) reveals the formation of stable cooperative clusters among international authors working on SWE. However, there has been limited collaboration between authors from different countries. Early on, authors like Mickaël Tanter were prolific in their publications. In recent years, however, a growing number of Chinese authors, including Xiaoyan Xie, Li Qiu, and others, have significantly increased their publication output and are gradually assuming more prominent roles in the field of SWE research.

### 3.5 Co-cited References Analysis

**Fig. (6):**
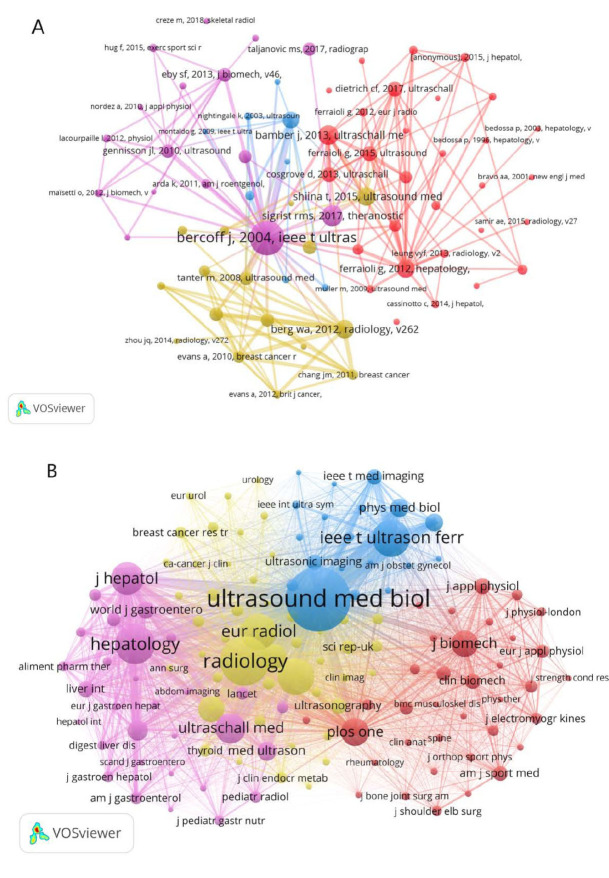
Co-citation network analysis of SWE references. (**A**) Network clustering diagram of co-cited references. The node size indicates the frequency of citation for each reference.The thickness of the lines between nodes represents the co-citation frequency of the references. The same color indicates that these references have a strong correlation. (**B**) Cluster diagram of major journals published by co-cited references. The same color indicates a strong correlation among the literature published by the journals.

We employed VOSviewer to conduct a co-citation network analysis of the references within our research dataset. The size of the nodes in the visualization corresponded to the frequency of citation for each reference. Notably, J. Bercoff’s seminal work from France, “Supersonic Shear Imaging: A New Technique for Soft Tissue Elasticity Mapping”, published in 2004, held the record for the most citations (652) (Table **4**). Following Bercoff, Rosa M.S. Sigrist’s team from the United States published an article in 2017 that has been cited 363 times. The visualization in Fig. (**6A**) illustrates that thicker lines between nodes signify a higher co-citation frequency for the references. We further conducted a cluster analysis of the references with high co-citation frequencies, which were primarily grouped into four clusters. These clusters were found in journals, such as Ultrasound in Medicine and Biology, Radiology, Hepatology, and Plos One (Fig. **6B**).

### 3.6 Keywords Analysis

**Fig. (7):**
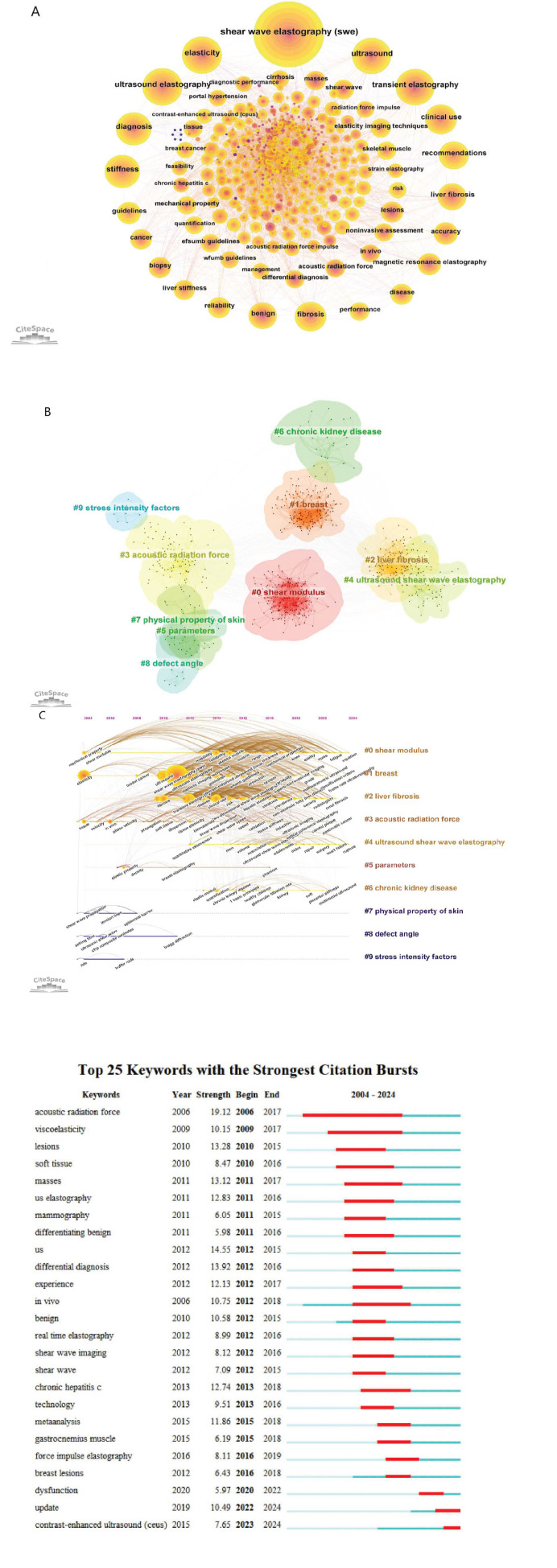
Visualization of keyword analysis. (**A**) The network of keywords. The size of the node represents the frequency of the keyword. (**B**) Cluster analysis of keywords. (**C**) Timeline view of the research on SWE. (**D**) Top 25 keywords with the strongest citation bursts.

Through the application of CiteSpace software, a visual analysis of the keywords extracted from the 3,606 papers related to SWE yielded a total of 806 distinct terms. As depicted in Fig. (**7A**), beyond the search terms and general descriptors, like “Shear Wave Elastography (SWE)”, “elasticity”, “ultrasound elastography”, and “ultrasound”, the top five most frequent keywords were “transient elastography”, “stiffness”, “diagnosis”, “fibrosis”, and “clinical use”. These keywords encapsulated the core themes of the research within the SWE domain. Relevance refers to how closely a keyword is related to the core topics of the analyzed field. In keyword clustering, relevance is assessed based on the frequency of keywords co-occurrence within the dataset and their contextual relationships in the text. Therefore, CiteSpace was utilized to categorize the identified keywords into 10 clusters based on their relevance (Fig. **7B**). The internal labels of each cluster are provided in Table **5**. The timeline graph (Fig. **7C**) charts the evolving trends of the keywords over time, and Fig. (**7D**) highlights the top 25 keywords with the strongest citation bursts, with “acoustic radiation force” exhibiting the highest burst intensity and duration. Since 2023, “contrast-enhanced ultrasound” has emerged as a new research hotspot within the SWE field.

## 4 DISCUSSION

In this bibliometric analysis, we have undertaken a comprehensive review of SWE research papers included in the WOSCC database over the past two decades (2004-2024). Our study has identified 3,606 relevant papers, reflecting the substantial growth and development of the field. The data showed a recent downward trend in the number of publications over the past two years, which may be attributed to the limitations of SWE [12, 13]. For example: (1) if the shear wave passes through a regular but non-uniform structure, it will be affected by significant anisotropy, leading to significant slowing of its speed when passing through multiple interfaces; (2) for individuals with BMI ≥ 30 kg/m^2^, the shear wave attenuates, leading to lower results. Despite these limitations, the overall trend remained upward. The number of published documents indicated that the research field and scope are still expanding, making ongoing research valuable and meaningful.

The study involved contributions from 70 countries, with China leading in terms of productivity and the United States demonstrating a higher scientific influence. Urban, Matthew W., *et al*. were among the most prolific authors of the Mayo Clinic institution, which was the leading representative in the United States. Their primary research focused on developing new imaging tools and methods to improve the diagnosis and treatment of soft tissue lesions [14]. Additionally, China had six of the top 10 most prolific institutions. Among them, the most prolific was Sun Yat-Sen University, indicating its position as an international leader in research in this field. Recently, the university evaluated the prognosis of patients with Acute-on-Chronic Liver Failure by combining SWE with other techniques [15]. This research has been reported to align with the future direction of SWE. The most frequently cited literature is highly influential in the academic research of this field. J. Bercoff *et al*. introduced the technology of supersonic shear imaging in 2004 [16], significantly influencing the field and becoming a cornerstone in the development of SWE. The second most-cited literature reviewed the value of ultrasound elastography in clinical applications [17]. Key publications by J. Bercoff, Rosa M.S. Sigrist, and J. Bamber have laid a solid foundation for future research in SWE.

The timeline view analysis indicated that early SWE research initially focused on mechanical properties, elasticity, shear modulus, and shear wave propagation. It showed that from 2004 to 2008, the principles and technology of SWE were the main research focus. The emergence of “breast cancer” as a keyword in 2008 suggested the adoption of SWE for disease diagnosis and evaluation. After SWE was first introduced in 2011, the focus shifted to keywords, such as “disease”, “clinical use”, “fibrosis”, “skeletal muscle”, “chronic kidney disease”, and “non-alcoholic fatty liver disease”. This indicates that the research focus gradually shifted from principles to the evaluation and classification of pathological tissues. Data analysis showed SWE to be mainly applied clinically in the diagnosis of liver diseases, breast lesions, thyroid nodules, and kidney, but research in areas, such as vessels, nerves, musculoskeletal system, and lung, is relatively limited (Table **6**). It holds certain significance for clinical guidance.

High-frequency keywords and burst terms have provided insights into future research directions. The most frequent keyword was “Transient Elastography (TE)”. In the early stages, it was generally believed that non-invasive liver stiffness assessment using TE is a reliable tool for detecting fibrosis and cirrhosis in patients with liver diseases [18, 19]. However, as research progressed, scholars increasingly found that for diagnosing significant liver fibrosis, the measured values fluctuated greatly, and the stage could not be accurately diagnosed [20, 21]. Subsequently, studies by Leung, Vivian Yee-Fong *et al*. found that across all stages of fibrosis, the accuracy of liver shear wave elastography was significantly higher than that of liver transient elastography [22]. Other studies found Two-dimensional Shear Wave Elastography (2D-SWE) to be more reliable than TE in detecting liver stiffness in patients with ascites [23]. This led to a downward trend in the frequency of TE use, with TE gradually being replaced by SWE.

The analysis of burst words revealed “acoustic radiation force” to be the keyword with the longest duration. As the imaging principle of SWE, acoustic radiation force first generates shear waves in tissue, then detects their propagation, and processes them to create a quantitative map of tissue elasticity [24]. The emission of the acoustic radiation force also slightly shifts the tissue at the focal point. The transducer then switches to imaging mode to detect the displacement by tracking the ultrasonic signal, referred to as a “spot”, to infer the tissue hardness [25]. Subsequently, Kathy Nightingale's team in the United States developed Acoustic Radiation Force Impulse Imaging (ARFI Imaging) based on this principle [26]. ARFI Imaging was then applied clinically and compared with SWE. Studies have shown both SWE and ARFI to be effective methods for evaluating liver fibrosis and not affected by ascites. However, SWE is more accurate than ARFI in diagnosing severe fibrosis (F = 2) [27-29]. Therefore, it is understandable that acoustic radiation force gained considerable attention during this stage.

The research value of CEUS in SWE-related fields has gained attention in recent years. A study by Wang Bin *et al*. found that combining CEUS and SWE can improve the sensitivity and accuracy of diagnosing benign and malignant thyroid nodules (types 4 and 5) co-existing with Hashimoto’s thyroiditis compared to using CEUS or SWE alone [30]. Additionally, combining CEUS and SWE has become increasingly important for early symptom monitoring in children with Crohn's disease [31], predicting the recurrence of hepatocellular carcinoma after curative treatment [32], and differentiating between benign and malignant conditions in pancreatic tumors, kidney diseases, liver cancer, and cervical lymph nodes [33-36]. Studies demonstrating the complementary nature of CEUS and SWE in improving the accuracy and sensitivity of disease diagnosis suggest that CEUS could potentially emerge as a prominent research hotspot within this field. Researchers utilized the Web of Science Core Collection (WOSCC) database and searched for relevant literature using “SWE and CEUS” and their synonyms. The number of publications per year is shown in Fig. (**8**), further illustrating the growing interest of researchers in this field.

Despite these findings, our study has involved certain limitations. Firstly, it was restricted to English-language publications in the SCI-E database of the WOSCC, which may have resulted in the omission of relevant studies available in other databases or languages. Secondly, due to the continuous updating of the database, the number of documents included may have not accurately reflected the total number of publications. For more comprehensive results, future research could explore additional databases, such as Medline, Scopus, and Google Scholar.

## CONCLUSION

Bibliometric analysis has afforded a systematic overview of the literature on SWE over the past two decades. The analysis has revealed an upward trend in the number of publications worldwide, with China and the United States leading the field in terms of both volume and collaborative efforts. The research interest in CEUS combined with SWE is on the rise, indicating promising prospects for the diagnosis of various diseases through the combined application of these two techniques. Given the current trajectory of development, it is anticipated that global research on SWE will continue to expand. The future research direction is expected to involve the ongoing exploration and expansion of clinical applications for multimodal ultrasound, which integrates SWE with other imaging modalities to enhance diagnostic accuracy and utility.

## Figures and Tables

**Fig. (5):**
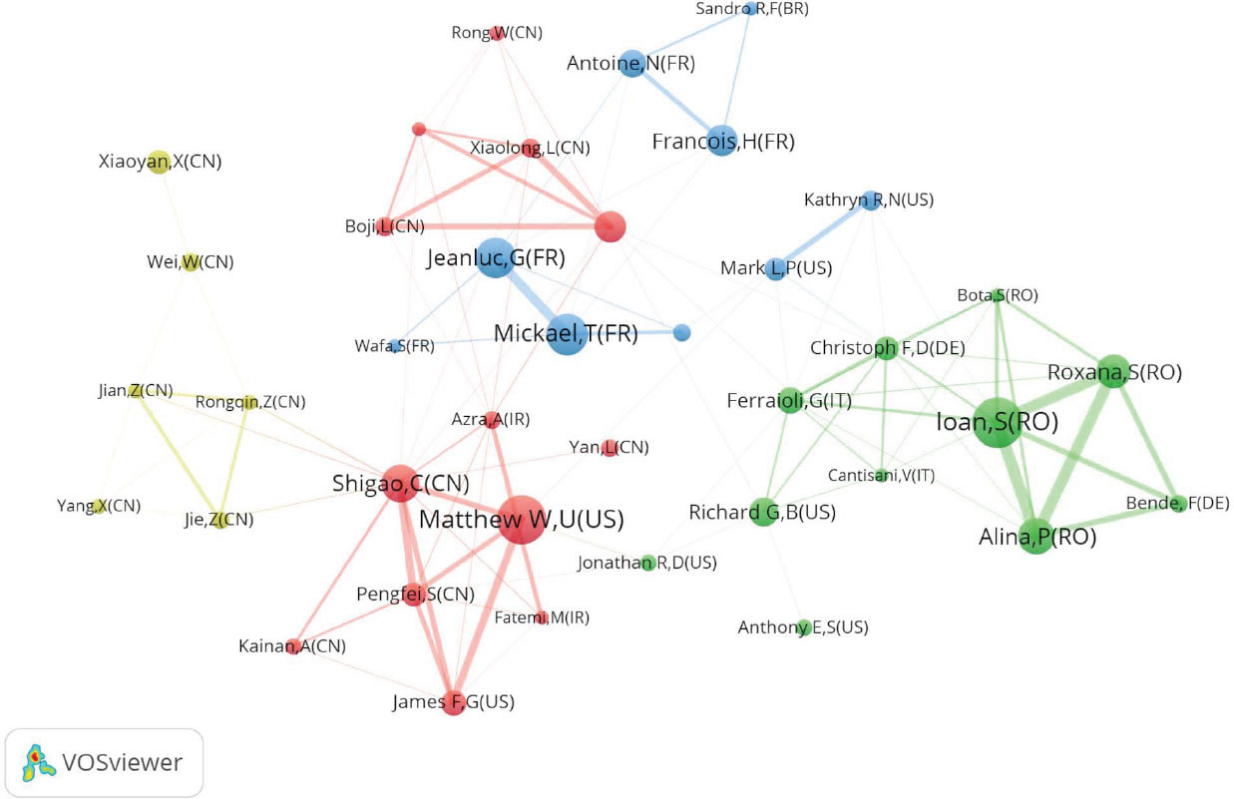
Collaboration network of authors. The node size indicates the number of publications; the link size refers to the cooperation intensity.

**Fig. (8):**
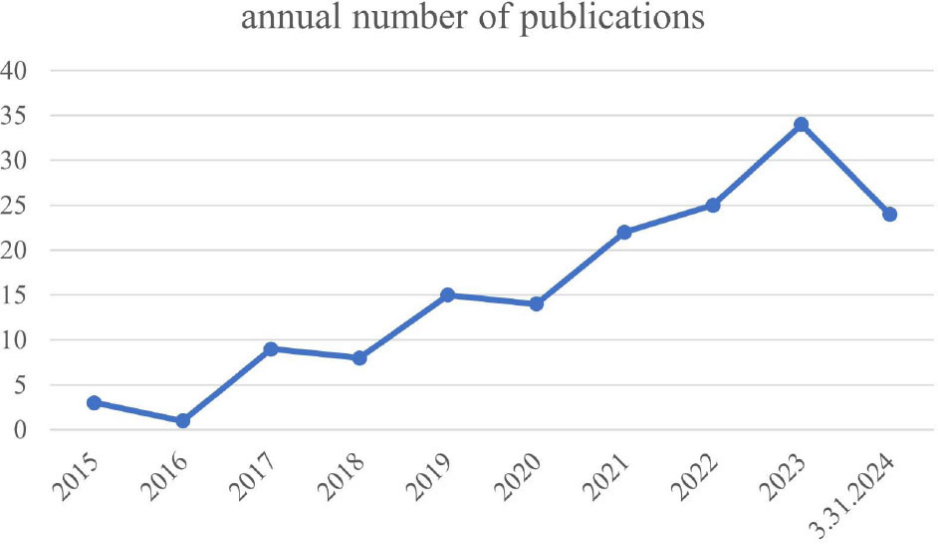
Annual publications on SWE and CEUS.

**Table 1:** Top 10 productive countries.

**Rank**	**Countries**	**Documents**	**(%)**	**Citations**	**Total Link Strength**	**Centrality**
1	China	993	27.54%	13539	243	0.01
2	USA	645	17.89%	16745	414	0.49
3	Japan	340	9.43%	5834	127	0.12
4	France	297	8.24%	11483	330	0.14
5	Turkey	295	8.18%	2424	20	0.06
6	South Korea	262	7.27%	6148	44	0
7	Germany	201	5.57%	6210	284	0.11
8	United Kingdom	165	4.58%	6701	251	0.19
9	Italy	153	4.24%	6774	215	0.08
10	Romania	107	2.97%	4001	143	0.03

**Note: **Centrality: the importance and influence of a node in a network. Total Link Strength: the sum of all connections between a node and other nodes, and it reflects the degree of closeness in the relationships between nodes.

**Table 2:** Top 10 productive institutions.

**Rank**	**Institutions**	**Country**	**Documents**	**Citations**
1	Sun Yat Sen University	China	130	2578
2	Mayo Clinic	USA	108	2961
3	Fudan University	China	67	1719
4	Sichuan University	China	66	1249
5	Capital Medical University	China	53	911
6	Shanghai Jiao Tong University	China	53	441
7	Seoul National University (SNU)	South Korea	53	1634
8	Kyoto University	Japan	52	1298
9	Tongji University	China	52	1110
10	Duke University	USA	51	1764

**Table 3:** Top 10 productive authors and top 10 co-cited authors with the highest citations.

**Rank**	**Author**	**Country**	**Documents**	**Citations**	**Total Link Strength**	**Rank**	**Co-cited Author**	**Count**
1	Sporea, Ioan	Romania	51	1854	116	1	Ferraioli, Giovanna	2832
2	Urban, Matthew W	USA	50	1068	58	2	Tanter, Mickael	2521
3	Tanter, Mickael	France	42	2521	46	3	Gennisson, Jean-Luc	2208
4	Gennisson, Jean-Luc	France	41	2208	38	4	Dietrich, Christiph F	2052
5	Ichihashi, Noriaki	Japan	40	690	50	5	Sporea, Ioan	1854
6	Chen, Shigao	China	38	1779	90	6	Chen, Shigao	1779
7	Popescu, Alina	Romania	37	716	100	7	Barr, Richard G	1641
8	Sirli, Roxana	Romania	35	717	99	8	Song, Pengfei	1586
9	Xu, Huixiong	China	32	653	60	9	An, Kai-nan	1192
10	Hug, Francois	France	32	1012	21	10	Cantisani, Vito	1121

**Note: **Count: the number of times co-cited authors are cited.

**Table 4:** Top 10 co-cited references.

**Rank**	**First Author**	**Title**	**Journal**	**IF**	**Year**	**Total Citations**
1	Bercoff, Jeremy	Supersonic shear imaging: a new technique for soft tissue elasticity mapping	IEEE Transactions onUltrasonics, Ferroelectrics, and Frequency Control	3.60	2004	652
2	Sigrist, Rosa	Ultrasound Elastography: Review of Techniques and Clinical Applications	Theranostics	12.40	2017	363
3	Bamber, Jeffrey	EFSUMB Guidelines and Recommendations on the Clinical Use of Ultrasound Elastography. Part 1: Basic Principles and Technology	Ultraschall in der Medizin	3.40	2017	348
4	Shiina, Tsuyoshi	WFUMB guidelines and recommendations for clinical use of ultrasound elastography: Part 1: basic principles and terminology	Ultrasound in Medicine & Biology”	2.489	2015	322
5	Berg, Wendie A	Shear-wave elastography improves the specificity of breast US: the BE1 multinational study of 939 masses	Radiology	19.70	2012	320
6	Ferraioli, Giovanna	Accuracy of real-time shear wave elastography for assessing liver fibrosis in chronic hepatitis C: A pilot study	Hepatology	14.0	2012	305
7	Sarvazyan, Armen P	Shear wave elasticity imaging: A new ultrasonic technology of medical diagnostics	Ultrasound In Medicine and Biology	2.90	1998	281
8	Ferraioli, Giovanna	WFUMB guidelines and recommendations for clinical use of ultrasound elastography: Part 3: liver	Ultrasound In Medicine and Biology	2.90	2015	251
9	Dietrich, Christoph F	EFSUMB Guidelines and Recommendations on the Clinical Use of Liver Ultrasound Elastography, Update 2017 (Long Version)	Ultraschall In der Medizin	3.40	2017	249
10	Cosgrove, David	EFSUMB guidelines and recommendations on the clinical use of ultrasound elastography. Part 2: Clinical applications	Ultraschall In der Medizin	3.40	2013	244

**Table 5:** Internal labels for each cluster keyword.

**Cluster-ID**	**Internal Labels**	**Size**
0	Shear modulus; muscle stiffness; muscle; skeletal muscle; liver fibrosis	187
1	Breast; breast cancer; thyroid nodule; liver fibrosis; benign	165
2	Liver fibrosis; liver stiffness; transient elastography; portal hypertension; cirrhosis	149
3	Acoustic radiation force; soft tissue; viscoelasticity; ultrasonic imaging; viscoelastic	101
4	Ultrasound shear wave elastography; skin; quantitative assessment; chronic obstructive pulmonary disease; familial mediterranean fever	59
5	Parameters; moduli; elastic property; poissons ratio; density	27
6	Chronic kidney disease; kidney; kidney transplantation; liver stiffness; elastic moduli	26
7	Physical property of skin; hemodialysis; shear wave propagation; aging; shear wave elastography	16
8	Defect angle; hydration; ultrasonic shear wave; test; azimuthal scanner	13
9	Stress intensity factors; inverse analysis; small closed crack; ultrasonic nondestructive evaluation; crack closing stress	8

**Table 6:** Clinical directions of SWE.

**Apply Directions**	**Documents**	**(%)**
Liver diseases	832	23.07%
Breast lesions	432	11.98%
Thyroid nodules	176	4.88%
Kidney	125	3.47%
Nerves	99	2.75%
Blood vessels	89	2.47%
Musculoskeletal	63	1.75%
Lung	35	0.97%

## Data Availability

The data and supportive information are available within the article.

## LIST OF ABBREVIATIONS

TLS: Total link strength
EFSUMB: European Federation of Ultrasound in Medicine and Biology
CEUS: Contrast-enhanced ultrasound
WOSCC: Web of Science Core Collection
SWE: Shear wave elastography
